# Severe morbidity after antiretroviral (ART) initiation: active surveillance in HIV care programs, the IeDEA West Africa collaboration

**DOI:** 10.1186/s12879-015-0910-3

**Published:** 2015-04-09

**Authors:** Yao Abo, Marcel Zannou Djimon, Eugène Messou, Eric Balestre, Martial Kouakou, Jocelyn Akakpo, Carin Ahouada, Nathalie de Rekeneire, François Dabis, Charlotte Lewden, Albert Minga, on behalf of the IeDEA West Africa Collaboration

**Affiliations:** Programme PAC-CI, Centre Hospitalier Universitaire (CHU) de Treichville, 18 BP 1954 Abidjan 18, Côte d’Ivoire; INSERM, Centre INSERM U897-Epidémiologie-Biostatistique, Bordeaux, France; University Bordeaux, ISPED, Bordeaux, France; Centre de Traitement Ambulatoire (CTA), Centre National Hospitalier Universitaire (CNHU), Cotonou, Benin; Centre de Prise en charge de Recherche et de Formation (Aconda-CePReF), Abidjan, Côte d’Ivoire; Université d’Abomey-Calavi, Cotonou, Bénin; Centre Médical de Suivi des Donneurs de Sang (CMSDS), Centre National de Transfusion Sanguine, Abidjan, Côte d’Ivoire

**Keywords:** Antiretroviral therapy, Severe morbidity, Ambulatory HIV care facilities, West Africa

## Abstract

**Background:**

The causes of severe morbidity in health facilities implementing Antiretroviral Treatment (ART) programmes are poorly documented in sub-Saharan Africa. We aimed to describe severe morbidity among HIV-infected patients after ART initiation, based on data from an active surveillance system established within a network of specialized care facilities in West African cities.

**Methods:**

Within the International epidemiological Database to Evaluate AIDS (IeDEA) - West Africa collaboration, we conducted a prospective, multicenter data collection that involved two facilities in Abidjan, Côte d’Ivoire and one in Cotonou, Benin. Among HIV-infected adults receiving ART, events were recorded using a standardized form. A simple case-definition of severe morbidity (death, hospitalization, fever > 38°5C, Karnofsky index < 70%) was used at any patient contact point. Then a physician confirmed and classified the event as WHO stage 3 or 4 according to the WHO clinical classification or as degree 3 or 4 of the ANRS scale.

**Results:**

From December 2009 to December 2011, 978 adults (71% women, median age 39 years) presented with 1449 severe events. The main diagnoses were: non-AIDS-defining infections (33%), AIDS-defining illnesses (33%), suspected adverse drug reactions (7%), other illnesses (4%) and syndromic diagnoses (16%). The most common specific diagnoses were: malaria (25%), pneumonia (13%) and tuberculosis (8%). The diagnoses were reported as syndromic in one out of five events recorded during this study.

**Conclusions:**

This study highlights the ongoing importance of conventional infectious diseases among severe morbid events occurring in patients on ART in ambulatory HIV care facilities in West Africa. Meanwhile, additional studies are needed due to the undiagnosed aspect of severe morbidity in substantial proportion.

**Electronic supplementary material:**

The online version of this article (doi:10.1186/s12879-015-0910-3) contains supplementary material, which is available to authorized users.

## Background

In December 2010, an estimated 5,640,000 Human Immunodeficiency Virus (HIV)-infected people were receiving Antiretroviral Treatment (ART) in Sub-Saharan Africa (SSA), which represented a 20% increase in ART coverage compared to a year earlier [1]. Despite the rapid increase in availability of antiretroviral drugs in SSA since 2004 [2], mortality and morbidity associated with HIV remain high among patients starting ART [3,4]. This high frequency of Acquired Immuno Deficiency Syndrome (AIDS)-related morbidity is due to late diagnosis of HIV infection and consequently leading to a late initiation of treatment [5].

According to the available literature, severe morbidity is common during the first months after ART initiation and remains high thereafter. Specifically, this severe morbidity is characterized by a high incidence of invasive bacterial diseases and tuberculosis [6,7]. Among SSA hospitalized HIV-positive patients, the most frequent causes of hospitalization are AIDS-defining conditions, tuberculosis and bacterial infections [8-10]. A high incidence of serious morbidity was recorded among HIV-infected adults followed up in routine conditions in SSA ambulatory care centers [11,12]. In addition to these infectious diseases, antiretroviral drugs expose patients to severe adverse events [13-15]. With the increase of life expectancy as the result of ART initiation, HIV-infected patients are more likely to present with non infectious diseases [16].

Available data on morbidity mainly come from research settings with advanced diagnostic capacities [3,4,7]. In SSA, causes of severe morbidity and mortality after ART initiation are poorly documented in health facilities implementing ART under routine circumstances, as data collection is still a challenge in those settings [17].

To fill this lack of data, the International epidemiological Database to Evaluate AIDS (IeDEA) was implemented in the West African region. It was launched to collect and harmonize data from multiple HIV⁄AIDS cohorts from industrialized and resource-limited countries. The IeDEA collaboration aims to address research questions in the field of HIV⁄AIDS care and treatment, especially in low-income countries, where most ART scale-up take place. The IeDEA West Africa is a prospective and observational multi-cohort collaboration established in 2006. It includes data from 8 countries with 15 centers for adult-patients infected with HIV on ART in Benin, Burkina Faso, Côte d’Ivoire, Guinea-Bissau, Mali, Nigeria, Senegal and Togo [18,19].

This collaboration could help address questions that have been poorly documented in this context, such as morbidity. Because of rapid changes in therapeutic strategies, a minimal morbidity surveillance in HIV care facilities of SSA is required [20-22].

The objective of our study was, to describe severe morbidity occurring among HIV-infected adult patients, receiving ART based on surveillance data collected prospectively under routine circumstances in HIV/AIDS care facilities in SSA.

## Methods

### Study design

The current study was conducted between December 2009 and December 2011, within the IeDEA network in West Africa [17,18] (see Additional file 1). For the purpose of this project, we conducted a prospective, multicenter ancillary study that involved three health care facilities. Among them, two were in Abidjan, Côte d’Ivoire: the Medical Center for the Monitoring of Blood Donors (CMSDS) and the Centre for Care, Research and Training (Aconda-CePReF). The other one was in Cotonou, Benin: the Outpatient Care Center of the National University Hospital (CTA/CNHU). All these three facilities are ambulatory HIV care centers that participated on a voluntary basis.

### Study population

The study population consisted of HIV-infected individuals, aged 18 and over, receiving ART in one of the three participating facilities. Those centers were chosen on the basis of a convenience sampling among the ambulatory care centers participating in the West Africa IeDEA collaboration.

### Definition of severe morbidity

We defined a severe event as the occurrence of one of the following events: death, hospitalization, fever (temperature above 385°C) or Karnofsky index < 70% in any patient on ART. Any patient with at least one of these signs was included in this study. The centers involved in this study were exclusively outpatient clinics. Therefore, deaths were reported by the patients’ siblings during telephone reminders made by the social workers. A standardized data collection form was used to record these events.

Physicians and other Health care workers (HCWs) (data collection technicians, social workers, nurses, pharmacy aides) from participating centers were trained to identify severe morbidity based on the four signs included in the definition. HCWs other than physicians, initiate a form by filling in the survey form with the sign(s) of severe morbidity experienced by the patients and their identification numbers. Physicians were responsible for completing the forms with clinical examination results. In case the physician noticed signs of severe morbidity by himself, he could initiate and fill the entire record*.*

Once the form was completed by the physician, additional testing might be requested to confirm the diagnosis. The final diagnosis was established by the physician in charge of the patient. This final diagnosis, either accurate or syndromic was chosen for the study. First, the diagnosis was accurate when it led to a specific disease and was based on additional testing. Then, the diagnosis was syndromic when it was mentioned as a sign and was not based on further investigation*.* Each diagnosis was classified by the physician according to the World Health Organization (WHO) clinical stage classification [23] and/or to the the Agence Nationale de Recherche sur le Sida et les hépatites Virales (ANRS) [24].

A physician was appointed at each center for the supervision of the study. He/she received all the forms daily, checked data completeness and the accuracy.

### Data collection

First, the physician in charge of the study at each center controlled the quality of the filled out survey forms. Next, documented severe events were subsequently coded according to a validated list of codes of the International Classification of Diseases, tenth revision (ICD-10) [25]. Then, the coded events were entered into a computer database and transferred from each health facility to the central database at the IeDEA West Africa coordination center.

### Main diagnostic procedures

Ambulatory care centers for HIV-infected patients need to provide them with, both routine follow-up laboratory examination and additional laboratory testing. The Table 1 gives a summary of the available tests.

**Table 1:** **Main diagnostic procedures in the three participating centers, IeDEA West Africa, December 2009 to December 2011**

**Centers**	**CMSDS**	**Aconda-CePReF**	**CTA/CNHU**
**Diagnostic procedures**			
**Monitoring routine tests**			
Full blood count	*	*	*
CD4 lymphocytes	*	*	*
Plasma urea and creatinine	*	*	*
Glycemia	*	*	*
**Additional tests for diagnosis of diseases**			
Thick film	*	**	***
Sputum examination	***	**	***
Radiography	***	**:	**:
Computed Tomography	***	**	***

*Available at the center and free of charge.

**Available on site but not free of charge.

***Not available at the center.

### Description of study findings

For each severe event, we described the main diagnosis, among the one, two or three diagnoses reported by the physician. If a patient had several diagnoses, we determined one main diagnosis by using the following priority order: first, WHO stage 4 opportunistic disease, second, WHO stage 3 opportunistic disease, third, other infection, fourth, other cancer, Fifth, WHO stage 4 cachexia, sixth, other disease and seventh, non specific event including syndromic diagnosis and non specific WHO stage 3 events (weight loss > 10%, chronic diarrhoea, persistent fever). We also described the total number of diagnoses and their percentage separately, using the total number of events as denominator.

### Statistical methods

Patient characteristics were described using the median and interquartile range (IQR) of continuous variables and the frequency distribution of categorical variables.

### Ethical approval

The IeDEA West Africa Collaboration obtained authorization from the Ethics committee “Comité de Protection des Personnes Sud-Ouest et Outre-mer III” in Bordeaux, France. Moreover, each site obtained authorization from its National Ethics committee: The “Comité National d’Ethique et de la Recherche (CNER)”, in Côte d’Ivoire and the “Comité National d’Ethique pour la Recherche en Santé (CNERS)”, in Benin. The physicians in charge of patients at each site obtained written informed consent from the patients included in this study.

## Results

Between December 2009 and December 2011, 978 HIV-infected adults followed up in three facilities of the IeDEA West Africa collaboration and presenting at least with one severe event were included in this study. Patients were mainly female (71%), with a median age of 39 years (IQR: 33–45). The cumulative follow-up period from ART initiation to the first severe event was 3465 person-years (PY) with a median of 23.1 months [IQR (3.4 to 54.7)] (Table 2). Among the 978 patients, 570 (58.3%) were followed up at Aconda-CePReF, 210 (21.5%) at CTA/CNHU and 198 (20.2%) at CMSDS. In these 978 patients, 1449 events occurred: 955 (65.9%) at Aconda-CePReF, 255 (17.6%) at CTA/CNHU and 239 (16.5%) at CMSDS (Table 2).

**Table 2:** **Characteristics at initiation of antiretroviral treatment (ART) and during follow-up (last visit) of HIV-infected adults in the three participating centers, IeDEA West Africa, December 2009 to December 2011**

**Characteristics**	**Total**		**Aconda-CePReF**		**CMSDS**		**CTA/CNHU**	
	**N = 978**		**n = 570**		**n = 198**		**n = 210**	
**At ART initiation**								
Sex, woman, n (%)	692	(71)	420	(74)	140	(71)	119	(57)
Age (years), median (IQR)	39	(33–45)	36	(31–44)	35	(31–41)	36	(31–42)
BMI (Kg/m^2^), median (IQR)	20.1	(17.9-22.7)	19.7	(17.6-22.2)	20.8	(18.7-23.7)	21.0	(18.8-24.6)
CD4 (/mm^3^), number, median (IQR)	137	(59–239)	136	(61–249)	157	(60–242)	108	(47–180)
Antiretroviral treatment, n (%)								
2NRTIs + 1NNRTIs	678	(69.3)	381	(66.8)	135	(68,2)	162	(77,1)
2NRTIs + 1PI	87	(9)	48	(8)	26	(13)	13	(6)
Other	213	(22)	141	(25)	37	(19)	35	(17)
Follow-up data*								
Total follow-up (months), median (IQR)	34.9	(16.3-67.1)	35.5	(14.6-66.2)	35.0	(18.2-54.1)	33.5	(16.9-76.6)
Follow-up until event (months), median (IQR) ((I(((IQR)	23.1	(3.4-54.6)	24.6	(2.6-55.5)	18.7	(3.5-43.1)	23.2	(5.8-66.2)
CD4 cell/mm^3^ median (IQR)	269	(103–467)	267	(102–453)	289	(124–517)	237	(73–468)
WHO stage, n (%)								
Stage 1 or 2	167	(17.1)	29	(5)	119	(60.2)	19	(9)
Stage 3	242	(24.7)	128	(22)	48	(24.2)	66	(31)
Stage 4	80	(8.2)	41	(7)	27	(13.6)	12	(6)

IQR = Interquartile Range.

BMI = Body Mass Index.

WHO = World Health Organisation.

ART = Antiretroviral Treatment.

Aconda-CePReF = Centre for Care, Research and Training.

CMSDS = Medical Centre for Monitoring Blood Donors.

CTA/CNHU = Outpatient Treatment Centre of the National University Hospital.

NNRTIs = Non Nucleoside Reverse Transcriptase Inhibitors.

PIs = Protease Inhibitors.

AZT = Zidovudine.

D4T = Stavudine.

3TC = Lamivudine.

*: close to the event: At the time of the morbid event.

The main diagnoses reported among these 1449 severe events were: non-AIDS-defining infection (32.6%), AIDS-defining illness (33.2%) with 23.3% in WHO stage 3 and 9.5% in WHO stage 4, suspected adverse drug reactions (7.1%), and finally non-infectious diseases and non-AIDS-defining illnesses (4.8%). In 16% of the cases, the diagnosis remained syndromic (Table 3). In this group, anemia (32%), fever (20%) and digestive disorders (13%) were the most common syndromic diagnoses. Detailed diagnoses reported for these severe events are presented in Table 4. Malaria (25%), pneumonia (13.4%) and tuberculosis (7.7%) were the most common diagnoses. During the study period, 180 patients (13%) died and among 80% of them, the cause of death could not be determined with precision (Table 4). The distribution of the 1449 severe events according to the circumstances of their identification was: regular or day hospitalization (41.5%), fever (29.8%), death (8.6%), Karnofsky index < 70% (4.5%), other reasons (16%).

**Table 3:** **Main** ^*^ **diagnoses of the 1449 events reported in HIV-infected adults receiving antiretroviral treatment, IeDEA West Africa, December 2009 to December 2011**

**Diagnosis classification, number (%)**	**Total 1449**		**Aconda-CePReF**		**CMSDS**		**CTA/CNHU**	
	**N = 1449**		**n = 955**		**n = 239**		**n = 255**	
**AIDS-defining events**								
WHO stage 4 events	137	(9.5)	68	(7.1)	31	(13)	38	(14.9)
WHO stage 3 events	338	(23.3)	214	(22.4)	24	(10)	100	(39.2)
WHO stage 2 events	6	(0.4)	5	(0.5)	0	(0)	1	(0,4)
**Non-AIDS-defining events**								
Non AIDS classifying infections	472	(32.6)	379	(39.7)	51	(21.3)	42	(16.5)
Side effects**	104	(7.2)	34	(3.6)	32	(13.4)	38	(14.9)
Other diagnosed diseases	63	(4.3)	35	(3.7)	12	(5)	16	(6.3)
Syndromic diagnoses***	228	(16.0)	160	(17.0)	55	(23.0)	13	(5.1))
**Death**	101	(7.0)	60	(6.0)	34	(14.2)	7	(3.0)

*: one diagnosis per event.

**: (anemia = 36, peripheral neuropathy = 33, toxiderma = 21, neutropenia = 5, lipodystrophy = 4, acute delusional psychosis = 2, hyperlactatemia = 2, hepatitis = 1).

***[anemia = 72, fever = 46, general signs (Asthenia, anorexia = 37), digestive signs (diarrhea, vomiting) = 30, other signs = 43].

**Table 4:** **Syndromic and definitive diagnoses** ^γ^ **according to WHO stages and ANRS degrees of severity, in HIV-infected adults receiving antiretroviral treatment, IeDEA West Africa, December 2009 to December 2011**

**Diagnoses, number**	**Total**		**WHO classification**		**ANRS degrees of severity**		Hospitalisation*	Death**
	**N = 1675**	**(%)**	**Stage 3**	**Stage 4**	**Degree 3**	**Degree 4**		
**Infectious events**								
**Parasitic events**								
Malaria	359	(24.8)			99	12	127	
Cerebral toxoplasmosis	24	(1.7)	0	24			15	3
Other parasitic events^‡^	9	(0.6)		3	1		2	
**Bacterial events**								
Pneumonia	194	(13.4)	194				79	2
Tuberculosis	111	(7.7)	45	66			43	4
ENT infection	26	(1.8)			2		3	
Severe bacterial infection	17	(1.2)	17				7	1
Other events^β^	12	(0.8)			2	2	3	
**Mycotic events**								
Oral candidiasis	138	(9.5)	133				8	
Other mycoses^£^	25	(1.7)		11			1	
**Infectious diarrhoea**	121	(8.4)			45	12	75	
**Skin infections**	16	(1.1)			1		5	
**Viral infections** ^**π**^	35	(2.4)		25	1		15	1
**Other infectious events** ^**α**^	27	(1,9)			5	4	10	
**Non infectious diagnosis**								
Peripheral neuropathy***	52	(3.6)			39	5	5	
Toxiderma	19	(1.3)			6	7	11	
Tumors	17	(1.2)		11		1	9	
Other non infectious diagnoses^Ψ^	46	(3.2)			8	17	13	1
**Non specific events**								
Anemia^†^	164	(11.3)			57	82	84	3
Fever	49	(3.4)		4	9	4	10	1
Diarrhoea	25	(1.7)		5	11	1	18	2
Other general signs^θ^	57	(3.9)			23	16	41	2
Other non specific^ф^	31	(2.1)			7	5	32	3
**Death**	**101**							

ENT = Ear Nose Throat.

γ Several diagnoses for a same event.

*Number of events with at least one day in day care.

** Number of death occurring after the event.

*** including 37 neuropathies secondary to ART initiation.

(%) Percentage calculated based on the number of events.

^†^including 54 anaemia secondary to a treatment.

^‡^6 ameibiasis, 2 pneumocystoses, 1 microsporidia.

^β^9 urinary infections, 2 appendicites, 1 cholecystitis.

^£^11 vaginal candidosis, 10 oesophagal candidosis, 3 digestive candidosis, 1 cryptococcal.

^π^21 HIV-related cachexia syndrome**,** 8 herpes zoster, 4 HIV-related encephalopathies, 2 herpes.

Tumors: 9 kaposi, 4 benign tumors, 2 unkown evolutive tumors, 1 cervical cancer, 1 Non-Hodgkin malignant Lymphoma.

^Ψ^ Cardio-vascular (6 arterial hypertension, 4 peripheral veins, 2 stroke), Renal (6 nephropathy, 6 renal failure), metabolic (5 abnormal fat distribution, 1 lactic acidosis, 1 diabetes), Genito-obstetric (two abortions, two other), Gastrointestinal (3 gastric or duodenal ulcers, 1 chronic pancreatitis, 7 other.

^α^7 genital infections, 6 osteoarticular infections, 6 broncho-pneumopathy, 5 meningitis, 2 stomatitis, 1 hepatitis.

^θ^19 asthenia, 21 dehydration, 17 under-nutrition.

^Ф^1 digestive disorders, 6 hemiplegia/tetraplegia, 6 psychiatric signs, 5 neutropenia, 2 liver signs, 11 other signs.

## Discussion

A prospective data collection system for severe morbidity was performed in three ambulatory HIV care facilities in West Africa. We collected standardized data from several patient contact points in each facility. Non-AIDS-defining infectious diseases and AIDS-defining diseases were the most frequent groups of morbid events observed in one-third of the patients. The most common specific diagnoses were malaria, pneumonia and tuberculosis. The diagnosis was defined as syndromic in one out of five cases*.*

This study showed the feasibility of routine collection of severe morbidity data among patients on ART based on: (i) the definition of reported signs of severe morbidity adapted to all staff and all care facilities and (ii) the definition and use of a simple data form. This organization involving all staff (data collection technicians, social workers, paramedics and physicians) and all services (reception desk, pharmacy, nursing, medical consultation, day care) had the advantage of being reproducible and affordable due to its integration within the existing system.

Regarding the WHO clinical stage 3 or 4 events, our results are consistent with those of previous studies [3,4] and could be explained by the late diagnosis of HIV infection or delayed ART initiation in this resource-limited setting [21]. Despite recent and repeated changes in the criteria for ART initiation in resource-limited countries [20,21,26], these changes were far from being fully implemented at the time of the study in most of these countries. Previous studies conducted in the context of the 2006 WHO criteria for ART initiation, have shown that patients started ART late with low CD4 [27]. This delay in ART initiation could explain the persistence of AIDS-defining illnesses after ART initiation. Enhanced and multiple opportunities of HIV testing are necessary to change this pattern of events.

The World Health Organization recently recommended to supply centers with rapid diagnostic tests for malaria [28]. This decision was not implemented in the participating centers during the study period. Indeed, among the three participating centers, only the CMSDS performed routine thick smears in suspected malaria cases at no cost to patients. Despite the high endemicity of malaria in the study area and the consistency of our results with those of some authors [29], we discussed the hypothesis of a possible overestimation of this diagnosis as confirmed by other studies [30,31]. Indeed one of the signs of serious illness was fever which is also one of the main signs of malaria. Some patients were not able to benefit from this testing due to financial constraints. Some of them could have been wrongly classified as malaria cases, thus overestimating the number of cases.

We also found that pneumonia as well as tuberculosis were the most common frequent AIDS-defining event while on ART*.* This result was also mentioned in previous studies in SSA [32,33], confirming the endemicity of these lung diseases in the study area at the current level of ART coverage [34,35].

Improvements have been made in the health care system in Africa more particularly in SSA. However, progress remains to be made*.* Among the challenges health care services deal with in Africa, underdeveloped infrastructures and weak healthcare systems are the most predominant [36]. As regards to HIV management, over 75% of expenses related to HIV care are faced by patients, and more than 50% of these expenses are allocated to the diagnosis and treatment of opportunistic infections) [37-39]. One of the consequences of these weaknesses is the difficulty to investigate the diagnoses of morbid events observed in patients. Therefore, we recommend strengthening the capacity of laboratories in HIV care facilities, by providing them with general laboratory testing in addition to efforts made to have antiretroviral drugs and specific HIV tests available in outpatient centers. Some of the events in this study were reported as syndromic diagnoses. Given the seriousness of some of these signs (fever, diarrhea), providing outpatient facilities with rapid diagnostic tests for malaria or conventional X-rays, should elucidate some of the diagnoses. Indeed, these general laboratory tests are part of the essential components of a basic package of HIV care and treatment [40,41]. Free access to those general laboratory testing in addition to the current free access to ART and specific HIV testing [42,43] will improve their accessibility.

The main strengths of this study are its prospective and multicenter design. First, a prospective study because for each patient’s symptom, all the information were documented according to a standardized procedure that minimized the risk of information bias. Then, a multicenter study, as it involved a large and unselected population of HIV-infected patients in care in the West African region. The use of a standardized data collection form contributed to ensure accuracy in data collection and limitation of variations within and among facilities. The involvement of almost all health care professionals at all levels and from all services was also instrumental in collecting the maximum number of events occurring during the study period.

Our study also had some limitations. The severity of a morbid event can be a reason for treatment discontinuation in HIV care centers. Thus, the methodology of our study, which consisted in capturing information about patients with severe morbidities at the three study facilities, is a limitation. Some patients suffering from severe events with restricted mobility and those with severe events lacking financial resources to travel to the facilities may have been excluded from this study, therefore leading to a selection bias. Another limitation is that specific signs of interest were identified a priori before taking the event into account. Some events may have been severe but without any of these predefined signs, thus leading to their inappropriate exclusion. In addition, due to the lack of resources, some patients might have restricted access to definitive diagnoses. Besides, some hospitalizations might have not been reported to us. All these biases may have contributed to an underestimation of severe morbidity.

In order to carry out an efficient intervention and deliver a more adequate service in a context of general weak health systems, further studies with access to diagnostic tests are needed to confirm the patterns observed in terms of syndromic diagnoses. Such studies could contribute to promote funding and the routine use of general laboratory tests to diagnose morbid events in resource-limited settings.

## Conclusion

Standardized and routine collection of severe morbidity data is possible in HIV/AIDS care facilities under routine circumstances. This study highlights the ongoing importance of common infectious diseases among severe morbid events occurring in patients on ART in national HIV care facilities in West Africa. Meanwhile, additional studies are needed due to the undiagnosed aspect of severe morbidity in substantial proportion.

## Additional file

Additional file 1:
**The IeDEA West Africa Collaboration Study Group.**
