# Supplementary material for: Severe morbidity after antiretroviral (ART) initiation: active surveillance in HIV care programs, the IeDEA West Africa collaboration
Source: BMC Infect Dis. 2015 Apr 9;15:176. doi: 10.1186/s12879-015-0910-3 (PMC4396560; doi:10.1186/s12879-015-0910-3)
Supplement: Additional file 1: — The IeDEA West Africa Collaboration Study Group. [file 12879_2015_910_MOESM1_ESM.doc]

**Additional file 1**

**The IeDEA West Africa Collaboration Study Group:**

**Participating sites** (*members of the **Steering Committee,** ^§^members of the **Executive Committee**):

***Benin, Cotonou:***

Adults: Djimon Marcel Zannou*, Carin Ahouada, Jocelyn Akakpo, Christelle Ahomadegbé, Jules Bashi, Alice Gougounon-Houéto, Angèle Azon-Kouanou, Fabien Houngbé, Jean Sehonou (CNHU Hubert Maga).

Pediatrics: Sikiratou Koumakpaï*^§^, Florence Alihonou, Marcelline d’Almeida, Irvine Hodonou, Ghislaine Hounhoui, Gracien Sagbo, Leïla Tossa-Bagnan, Herman Adjide (CNHU Hubert Maga).

***Burkina Faso:***

Adults: Joseph Drabo*, René Bognounou, Arnaud Dienderé, Eliezer Traore, Lassane Zoungrana, Béatrice Zerbo (CHU Yalgado*,* ***Ouagadougou***), Adrien Bruno Sawadogo*^§^, Jacques Zoungrana, Arsène Héma, Ibrahim Soré, Guillaume Bado, Achille Tapsoba (CHU Souro Sanou, ***Bobo Dioulasso***)

Pediatrics: Diarra Yé*, Fla Kouéta, Sylvie Ouedraogo, Rasmata Ouédraogo, William Hiembo, Mady Gansonré (CH Charles de Gaulle*,* ***Ouagadougou***).

***Côte d’Ivoire, Abidjan:***

Adults: Eugène Messou*, Joachim Charles Gnokoro, Mamadou Koné, Guillaume Martial Kouakou, (ACONDA-CePReF); Clarisse Amani Bosse*, Kouakou Brou, Achi Isidore Assi (ACONDA-MTCT-Plus); Henri Chenal*, Denise Hawerlander, Franck Soppi (CIRBA); Albert Minga*, Yao Abo, Jean-Michel Yoboue (CMSDS/CNTS); Serge Paul Eholié*^§^, Mensah Deborah Noelly Amego, Viviane Andavi, Zelica Diallo, Frédéric Ello, Aristophane Koffi Tanon (SMIT, CHU de Treichville), Serge Olivier Koule*, Koffi Charles Anzan, Calixte Guehi (USAC, CHU de Treichville);.

Pediatrics: Edmond Addi Aka*, Koffi Ladji Issouf, Jean-Claude Kouakou, Marie-Sylvie N’Gbeche, (ACONDA-CePReF); Touré Pety*, Divine Avit-Edi (ACONDA-MTCT-Plus); Kouadio Kouakou*, Magloire Moh, Valérie Andoblé Yao (CIRBA); Madeleine Amorissani Folquet*, Marie-Evelyne Dainguy, Cyrille Kouakou, Véronique Tanoh Méa-Assande, Gladys Oka-Berete, Nathalie Zobo, Patrick Acquah, Marie-Berthe Kokora (CHU Cocody); Tanoh François Eboua*, Marguerite Timité-Konan, Lucrèce Diecket Ahoussou, Julie Kebé Assouan, Mabéa Flora Sami, Clémence Kouadio (CHU Yopougon).

***Ghana, Accra:***

Pediatrics: Lorna Renner*^§^, Bamenla Goka, Jennifer Welbeck, Adziri Sackey, Seth Ntiri Owiafe (Korle Bu TH).

***Guinea-Bissau:***

Adults: Christian Wejse*^§^, Zacarias José Da Silva*, Joao Paulo (Bandim Health Project), The Bissau HIV cohort study group: Amabelia Rodrigues (Bandim Health Project), David da Silva (National HIV program Bissau), Candida Medina (Hospital National Simao Mendes, Bissau), Ines Oliviera-Souto (Bandim Health Project), Lars Østergaard (Dept of Infectious Diseases, Aarhus University Hospital), Alex Laursen (Dept of Infectious Diseases, Aarhus University Hospital), Morten Sodemann (Dept of Infectious Diseases, Odense University Hospital), Peter Aaby (Bandim Health Project), Anders Fomsgaard (Dept. of Virology, Statens Serum Institut, Copenhagen), Christian Erikstrup (Dept. of Clinical Immunology), Jesper Eugen-Olsen (Dept. of Infectious Diseases, Hvidovre Hospital, Copenhagen).

***Guinea:***

Adults: David Leuenberger*, Jean Hebelamou^§^ (Centre Medical Macenta)

***Mali, Bamako:***

Adults: Moussa Y Maïga*^§^, Fatoumata Fofana Diakité, Abdoulaye Kalle, Drissa Katile (CH Gabriel Toure), Hamar Alassane Traore*, Daouda Minta*, Tidiani Cissé, Mamadou Dembelé, Mohammed Doumbia, Mahamadou Fomba, Assétou Soukho Kaya, Abdoulaye M Traoré, Hamady Traoré, Amadou Abathina Toure (CH Point G).

Pediatrics: Fatoumata Dicko*, Mariam Sylla, Alima Berthé, Hadizatou Coulibaly Traoré, Anta Koïta, Niaboula Koné, Clémentine N'Diaye, Safiatou Touré Coulibaly, Mamadou Traoré, Naïchata Traoré (CH Gabriel Toure).

***Nigeria:***

Adults: Man Charurat* (UMB/IHV), Vivian Kwaghe*^§^, Samuel Ajayi, Georgina Alim, Stephen Dapiap, Otu (UATH, ***Abuja***), Festus Igbinoba (National Hospital ***Abuja***), Okwara Benson*, Clément Adebamowo*, Jesse James, Obaseki, Philip Osakede (UBTH, ***Benin*** ***City***), John Olasode (OATH, ***Ile-Ife***).

***Senegal, Dakar:***

Adults: Moussa Seydi*^§^, Papa Salif Sow, Bernard Diop, Noël Magloire Manga, Judicael Malick Tine^§^, Coumba Cissé Bassabi (SMIT, CHU Fann),

Pediatrics: Haby Signate Sy*, Abou Ba, Aida Diagne, Hélène Dior, Malick Faye, Ramatoulaye Diagne Gueye, Aminata Diack Mbaye (CH Albert Royer).

***Togo, Lomé:***

Adults: Akessiwe Patassi*^§^, Awèrou Kotosso, Benjamin Goilibe Kariyare, Gafarou Gbadamassi, Agbo Komi, Kankoé Edem Mensah-Zukong, Pinuwe Pakpame (CHU Tokoin/Sylvanus Olympio).

Pediatrics: Koko Lawson-Evi*^§^, Yawo Atakouma, Elom Takassi, Améyo Djeha, Ayoko Ephoévi-gah, Sherifa El-Hadj Djibril (CHU Tokoin/Sylvanus Olympio).

**Executive Committee*:** François Dabis (Principal Investigator, Bordeaux, France), Emmanuel Bissagnene (Co-Principal Investigator, Abidjan, Côte d’Ivoire), Elise Arrivé (Bordeaux, France), Patrick Coffie (Abidjan, Côte d’Ivoire), Nathalie de Rekeneire (Bordeaux, France), Didier Ekouevi (Abidjan, Côte d’Ivoire), Antoine Jaquet (Bordeaux, France), Valériane Leroy (Bordeaux, France), Charlotte Lewden (Bordeaux, France), Annie J Sasco (Bordeaux, France).

**Operational and Statistical Team:** Jean-Claude Azani (Abidjan, Côte d’Ivoire), Eric Balestre (Bordeaux, France), Serge Bessekon (Abidjan, Côte d’Ivoire), Sophie Karcher (Bordeaux, France), Jules Mahan Gonsan (Abidjan, Côte d’Ivoire), Jérôme Le Carrou (Bordeaux, France), Séverin Lenaud (Abidjan, Côte d’Ivoire), Célestin Nchot (Abidjan, Côte d’Ivoire), Karen Malateste (Bordeaux, France), Amon Roseamonde Yao (Abidjan, Côte d’Ivoire).

**Administrative Team:** Abdoulaye Cissé (Abidjan, Côte d’Ivoire), Alexandra Doring^§^ (Bordeaux, France), Adrienne Kouakou (Abidjan, Côte d’Ivoire), Guy Gneppa (Abidjan, Côte d’Ivoire), Elodie Rabourdin (Bordeaux, France), Jean Rivenc (Pessac, France).

**Consultants/ Working Groups:** Xavier Anglaret (Bordeaux, France), Boubacar Ba (Bamako, Mali), Renaud Becquet (Bordeaux, France), Juan Burgos Soto (Bordeaux, France), Jean Bosco Essanin (Abidjan), Andrea Ciaranello (Boston, USA), Sébastien Datté (Abidjan, Côte d’Ivoire), Sophie Desmonde (Bordeaux, France), Jean-Serge Elvis Diby (Abidjan, Côte d’Ivoire), Geoffrey S.Gottlieb* (Seattle, USA), Apollinaire Gninlgninrin Horo (Abidjan, Côte d’Ivoire), Julie Jesson (Bordeaux, France), Serge N'zoré Kangah (Abidjan, Côte d’Ivoire), David Meless (Abidjan, Côte d’Ivoire) , Aida Mounkaila-Harouna (Bordeaux, France), Camille Ndondoki (Bordeaux, France), Caroline Shiboski (San Francisco USA), Boris Tchounga (Abidjan, Côte d’Ivoire), Rodolphe Thiébaut (Bordeaux, France), Gilles Wandeler (Dakar, Senegal).

**Funding:** The National Cancer Institute (NCI), the Eunice Kennedy Shriver National Institute of Child Health & Human Development (NICHD) and the National Institute of Allergy and Infectious Diseases (NIAID) of the U.S. National Institutes of Health (NIH), as part of the International Epidemiologic Databases to Evaluate AIDS (IeDEA) under Award Number U01AI069919. The content is solely the responsibility of the authors and does not necessarily represent the official views of the National Institutes of Health.

**Coordinating Center:** ISPED, Univ Bordeaux Segalen, Bordeaux, France

**Regional Office:** PAC-CI, Abidjan, Côte d’Ivoire

**Methodologic Support**: MEREVA, Bordeaux, France

**Website:** http://www.mereva.net/iedea
